# Cancer of the cervix in Zaria, Northern Nigeria

**DOI:** 10.3332/ecancer.2011.219

**Published:** 2011-08-19

**Authors:** OA Oguntayo, M Zayyan, AOD Kolawole, SA Adewuyi, H Ismail, K Koledade

**Affiliations:** 1 Department of O&G (Oncology Unit), Ahmadu Bello University Teaching Hospital, Zaria, Nigeria; 2 Radiotherapy and Oncology Centre, Ahmadu Bello University Teaching Hospital, Zaria, Nigeria

## Abstract

**Objective:**

Carcinoma of the cervix is still the most common gynecological malignancy among women in the developing nations. The purpose of this study is to review the pattern of carcinoma of the cervix in Zaria, Northern Nigeria.

**Method:**

This is a retrospective study of patients seen at the Gynecologic oncology unit of Ahmadu Bello University Teaching Hospital, Zaria, Nigeria between November 2005 and November 2009.

**Results:**

A total of 406 gynecological cancers were identified during the period under review. Carcinoma of the cervix accounted for 65.7 % (267) of histologically confirmed gynecological cancers. Most of the patients were married 265 (99.2 %) and 40% were in the second order of marriage; 57.1% of these women were in a polygamous setting. Two hundred and two (75.6 %) patients fell in the 40Œ69 year age bracket, with a mean age of 44.5 years. The disease appears to be associated with high parity (range of 0Œ14); grand multiparous patients constituted 145 (68.3%) of the cases. Abnormal vaginal bleeding (219 patients: 82 %), offensive vaginal discharge (120 patients: 44.9%) and post-coital bleeding (56 patients: 20.9%) were the most common symptoms. About 78% of the patients had advanced disease, stage III disease being the commonest stage accounting for 159 (59.5 %). Fifty-six (21%) of these patients presented with vesico-vaginal fistula.

**Conclusion:**

This study demonstrates that in the northern part of Nigeria 65.7% of all gynecological cancers are carcinoma of the cervix. This high percentage appears to be connected with some detrimental sociocultural practices, such as early onset of sexual activity, which should be addressed. More emphasis should be given to screening programs for women in under-developed countries.

## Introduction

Modern screening techniques mean that carcinoma of the cervix is a preventable cancer of our time [1]. A review of the literature indicates that the incidence and prevalence of the disease has reduced significantly in developed countries compared to developing countries [2]. This finding is not unconnected with the use of screening methods which is available and affordable in those environments.

In our own environment we noticed an increase in the incidence and prevalence of the disease and also that our patients present late with a variety of complications [1]. We have observed that there is no report on the enormity of this problem in Nigeria and so we undertook this retrospective study in our center, which is actually the center of excellence in oncology in Nigeria.

Understanding the degree of the problem will go a long way to influence the policy on health both locally and internationally.

Worldwide, cervical carcinoma is the second commonest female malignancy [3]. It is responsible for 300,000 deaths annually worldwide and over 500,000 new cases are reported each year [4–7]. Eighty percent of these new cases come from developing nations and as such it is the leading cause of cancer death in women in these countries.

The choice of treatment depends on the clinical stage of the disease and the availability of treatment facilities. In view of the paucity of radiotherapy machines, surgery combined with adjuvant chemotherapy is usually offered in most centers in Nigeria. In Zaria there is a radiotherapy machine but it has a high patient load and so the time interval between diagnosis and treatment is considerably long.

The high incidence of this disease in developing nations needs to be reduced [8] and so better treatment methods are urgently required. The triple approach (surgery, radiotherapy and chemotherapy) for treating cancer of the cervix is long overdue in these countries.

Further the level of awareness of cervical screening is low and even worse is the level of uptake; at present the level of uptake of 7.1% (60) and so no significant impact will be made on the incidence of cervical cancer with such a small percentage of individuals undergoing screening. Only 270 patients were screened as routine screening in 5 years [9]. The motivation for this study is that cervical carcinoma in developing nations needs to be addressed urgently and managed as such.

## Materials and methods

The study was conducted in Ahmadu Bello University Teaching Hospital, Department of Obstetrics and Gynaecology, Zaria. It was a retrospective study of all cancers of the cervix presenting at A.B.U. Teaching Hospital between 11 November 2005 and 10 November 2009. All the cervical cancers reviewed were confirmed by histological examination.

Clinical staging (FIGO Staging) of all cases was performed, under anesthesia, by senior registrars (associate fellows) or consultant gynecologists. Social classification was based on patients’ educational status and their husbands’ occupation (adopted method of Olusanya *et al*).

The excel statistical package was used for statistical analysis.

The analysis was based on descriptive statistics, using means, medians, modes, percentages and ratio standard deviation. The results were statistically significant when the *p*-values were ≤0.05. We also applied demographic representations.

## Results

A total of 406 cases of gynecological cancers were seen and managed. Two hundred and sixty-seven (65.7%) were primary cervical tumors histologically confirmed, making it the leading gynecological cancer.

**Figure 1:**
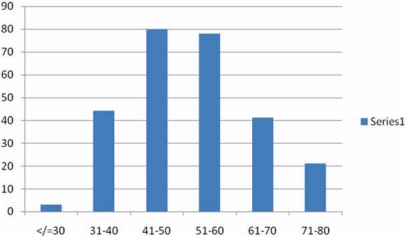
Age distribution.

The age range of the patients was from 19–80 years. The mean age was 44.5 years with 75.6% of the patients within the 41–60 year age bracket. The peak incidence was in the 41–50 year age group (see Figure 1). Two hundred and sixty five (99.2%) were married and 202 (75.7%) were of low socioeconomic status. It is important to observe, that none of these patients had ever heard of pap smear or had used it as a screening tool in the past.

The disease is more prevalent in women of high parity, the highest prevalence was in women with more than 5 pregnancies which represented 51% of the patients (136 cases) (Table 1).

**Figure 2:**
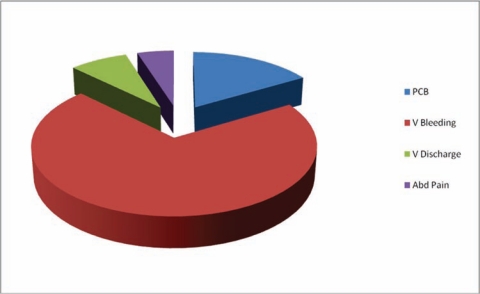
Symptoms at presentation.

The presenting symptoms are highlighted in Figure 2. Most of the patients had more than one symptom at presentation. Irregular vaginal bleeding was the commonest, accounting for 189 (70.8%) of the cases. Others include post-coital bleeding (16.5%), Vaginal discharge (7.8%) and abdominal pain (4.8%). Clinical staging of all cases were performed under anesthesia by Senior Registrars (associate fellows) or Consultant Gynecologists using the FIGO Staging.

Two hundred and sixty two (98%) presented with advanced stage of the disease (stage IIb – IVb). Only five (2%) presented early. We observed that stage III was the commonest accounting for 153 (57.3%) of cases (see Table 2). The 3 main histological types were found but the Squamous cell carcinoma constituted the majority 253(95%), while the Adenocarcinoma and Adenosquamous accounted for only 14 (5%) cases.

**Figure 3:**
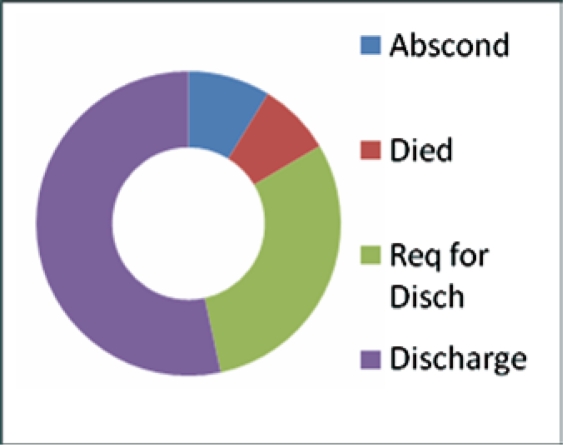
Outcome of management of cervical cancer.

The main modalities of treatment were radiotherapy, chemotherapy, chemo-radiation therapy and palliative care. Our main challenge was severe hemorrhage, occurring in 75 (18.5%) patients, in which case a hemostatic dose of radiotherapy was used. Eighty percent of our study population underwent a transfusion with between 2–7 units of blood. As a result of late stage presentation obstructive uropathy was observed in 17 (4.2%) cases, this poses a dilemma in the management of the disease. Other challenges included 20 (4.9%) patients with retroviral infections, some also had uremia, vesico-vaginal fistula and recto-vaginal fistula. The absence of funds and complications resulting in hemorrhagia were important factors affecting treatment completion. In fact 30.1 % of patients requested to be discharged from treatment for lack of funds, 8.74% absconded and 7.76% died either in the early days of treatment or even before commencement. While 53.4% were able to complete their treatment, only 5.2% were alive at five years (see Figure 3).

## Discussion

Carcinoma of the cervix still remains the leading cause of gynecological cancers in Northern Nigeria, accounting for 65.7% of all gynecological cancers. This high incidence was also observed in Ibadan and Maiduguri (Nigeria) with 62.7% and 72.6% respectively [12,14,15].

The incidence of carcinoma of the cervix is estimated to be 8–10/100,000 per year [11]. The incidence differs from one [12–15] place to another with a significant gap between the developing and developed nations. The fact still remains that it is the commonest gynecological cancer in the developing nations [16,17]. The reason for this high incidence is the lack of affordable and accessible screening facilities. Also illiteracy remains a major contributing factor limiting the reduction of this incidence [18,19] as our poor health decision making can only be explained by lack of knowledge. We also need to address our health seeking behavior, how can we justify medical practitioners who do not utilize available cancer screening facilities? A study carried out in Eastern Nigeria showed that the level of awareness of cervical screening is low and even worse is the level of uptake. The level of awareness was 52.8%, while only 7.1 % had ever done the test [20]. At the present level of uptake no significant impact will be made on the incidence of cervical cancer in developing countries.

The age distribution is similar to that reported by other centers [20,21]. The peak age incidence for this study is 45 years, similar to that reported by Rafindadi *et al* [14] and Ijaiya *et al* [25] and confirming that it is a disease of women of child-bearing age. The risk of developing cancer of the cervix was highest among grandmultiparous women (parity of 6 and above) which contitutes 63% of the study population. In fact other studies carried out in Africa have demonstrated an association between high number of deliveries and incidence of cervical cancer [19–21,25] and this study is confirmatory. This could be associated with the increased risk of exposure to the HPV virus in this group of women.

The commonest symptom at presentation was abnormal vaginal bleeding as seen in 82% of cases, though most of them had more than one symptom at presentation. This finding is similar to what was found in Ijaiya *et al* [25]. The stage distribution of the disease in our center is not different from what is obtainable elsewhere i.e. 78% of the patient presenting late (stage IIb–IVb) to the hospital [17,18]. Stage III carcinoma of the cervix was the most common stage seen at our center accounting for 59.5% of cases. This figure is similar to the experience in Ghana [19]. This to a large extent affects the management as well as the prognosis of the disease. It is surprising that in the study none of our patient presented with stage O or I, this is similar to Adewuyi report [24].

Squamous cell carcinoma was the commonest histological type encountered, accounting for 95% of cases seen. This finding is very similar to those of Ilorin [25] who had 85.7% and Maiduguri [12] with 92%.

This study has clearly demonstrated that cancer of the cervix is a serious problem for developing nations. The high incidence observed in this study is comparable to that of Ibadan (Nigeria) [15], and other developing nations such as Ghana (Kumasi) [19] and Iran [26]. This is saddening as it is a highly preventable cancer. Nigeria must wake up to it’s responsibilities and start education programs for adolescent girls, advocacy, enlightenment, mobilization and empowerment.

## Figures and Tables

**Table 1:** Parity distribution.

**Parity**	**Frequency**	**%**
0	5	1.8
1–5	94	35.2
6–10	136	51
>10	32	12
Total	267	100

**Table 2:** The clinical staging of the disease.

**Stage**	**Frequency**	**%**
I a&b	0	0
II a	5	1.87
II b	34	12.7
III a	31	11.6
III b	153	57.3
IV a	44	16.56
Total	267	100
